# Dental Plaque Removal with Two Special Needs Toothbrushes in Patients with Down Syndrome: A Parallel-Group Randomised Clinical Trial of Efficacy

**DOI:** 10.3290/j.ohpd.b3630331

**Published:** 2022-11-30

**Authors:** Hytham N. Fageeh, Manawar A. Mansour, Hammam I. Fageeh, Abdulkareem Hummadi, Turki Khurayzi, Khalil Marran, Naif Alqunfuthi, Shankargouda Patil

**Affiliations:** a Associate Professor, Department of Preventive Dental Science, College of Dentistry, Jazan University, Jazan, Saudi Arabia. Conceptualisation, original draft preparation, project administration, supervision, formal analysis.; b Assistant Professor, Department of Prosthetic Dental Sciences, College of Dentistry, Jazan University, Jazan, Saudi Arabia. Methodology, data curation, statistical analysis, resources, wrote, reviewed and edited the manuscript.; c Assistant Professor, Department of Preventive Dental Science, College of Dentistry, Jazan University, Jazan, Saudi Arabia. Conceptualisation, original draft preparation, project administration, supervision, formal analysis.; d Dentist, General Dentistry, College of Dentistry, Jazan University, Jazan, Saudi Arabia. Clinical examination and data collection.; e Adjunct Faculty, College of Dental Medicine, Roseman University of Health Sciences, South Jordan, Utah, USA. Study design, drafted and reviewed the manuscript.

**Keywords:** bleeding index, customised brush, Down syndrome, microbial contamination, plaque index, special needs toothbrush

## Abstract

**Purpose:**

To compare the effectiveness of two varieties of special needs toothbrushes in terms of dental plaque removal and bacterial contamination vs a conventional toothbrush in patients with Down syndrome.

**Materials and Methods:**

This single-blinded, two-group, randomised clinical trial included 16 patients diagnosed with Down syndrome (age 6–15 years) from various special needs centers located in the Jazan Province of Saudi Arabia. The patients were randomly allocated to two groups based on the type of special needs toothbrush provided (Collis Curve or superfine nano). The plaque and bleeding indices of the patients in both groups were measured at baseline (T_0_) and both groups were initially given a conventional toothbrush to use for four weeks. After this period, the plaque and bleeding indices were re-evaluated (T_1_). The patients were instructed to use the special needs toothbrush for 4 weeks, after which the periodontal indices were re-evaluated (T_2_). Microbial contamination on the bristles of the special needs brushes was evaluated at T_2_.

**Results:**

No notable changes in the mean plaque and bleeding indices were observed between the two groups at each visit; however, statistically significant reductions were noted between visits in both groups (p < 0.05). The CFU scores in cultures from the Collis Curve toothbrush bristles (1411.5 ± 541.1) were higher than those obtained from the superfine nano-toothbrush bristles (1118.3 ± 423.9), but without statistically significant differences.

**Conclusion:**

The findings indicate that the use of special needs toothbrushes can statistically significantly improve the gingival health status in individuals with Down syndrome in terms of both resolution of periodontal inflammation and reduction of plaque accumulation.

Down syndrome is a genetic condition that causes intellectual disability as a result of abnormal disjunction of chromosomes at the time of cell division, leading to a trisomy (an extra chromosome number 21).^12,28^ Each year, one out of every thousand children born worldwide is believed to have Down syndrome. In Saudi Arabia, its incidence is estimated to be 1 in every 554 live births.^35^ Consanguineous marriages, a prevalent practice in Saudi Arabia, are a predisposing risk factor.^14^

Individuals with Down syndrome are specifically predisposed to orofacial maladies such as periodontitis and occlusal disharmony, with a large tongue in a small oral cavity.^8,16^ Young patients (<35 years) with Down syndrome have an increased prevalence (58%–96%) of gingival and periodontal inflammation.^32,34^ The deterioration of periodontal health due to difficulty in oral hygiene maintenance by affected patients is a result of physical impairment and restricted manual dexterity due to the underlying neurological deficits.^20,29^ The suboptimal periodontal status in Down syndrome individuals is believed to be due to the immune system being compromised (decline in the level of T-lymphocytes), poor hygienic practices, delicate periodontal ligament tissue, premature aging, and improperly functioning masticatory apparatus.^19,22^ The other reported orofacial features in the syndrome that deserve mention include hypoplastic and supernumerary teeth, abnormal eruption orientations, clenching, ectopically erupted teeth, enlarged tongue, palatal vault, prognathic mandible, apertognathia, tongue with multiple fissures, angular cheilitis, small permanent teeth and large deciduous teeth.^21,46^ Additionally, the tooth phenotype affected results in discoidal frontal teeth, absent/shortened crestal margins, and furrowed occlusal tops in the posterior teeth. The prevalence of caries was reported to be high in these syndromic patients in Saudi Arabia.^4^

Oral hygiene is affected in patients with Down syndrome owing to the difficulty in hand-eye coordination, inadequate incentive, and difficulties in maintaining good dental hygiene practices using classical toothbrushes.^5^ Their lack of dexterity and fine motor coordination profoundly affect their oral health. However, caries and periodontal disease can be prevented by controlling the growth of the supragingival biofilm through mechanical plaque control measures such as toothbrushing.^45^ The etiological factors responsible for the relative lack of success of mechanical supra-gingival plaque control – along with adjunctive procedures such as scaling and root planing (SRP) to avoid periodontal disease in Down syndrome – may include a compromised immune system, characterised by low immune cell count and reduced vascular responses. A recent study outlined a treatment strategy for Down syndrome individuals through a monthly dental care and prevention program, including supra and sub-gingival SRP. This was effective in improving deteriorated periodontal conditions.^40^ This technique may be effective in compliant Down syndrome subjects, but would be challenging for most patients in the absence of general anesthesia/sedation.

Oral and dental hygiene promotional campaigns for special-need individuals are effective.^17^ Caregivers are given directives regarding dental hygiene to improve the oral health of their charges, which can lead to a decline in biofilm production and more conscientious manual brushing. The oral and periodontal condition of patients with intellectual disabilities is jeopardised to a greater degree than that of their age- and gender-matched contemporaries without intellectual disabilities. It is laborious, cumbersome, and difficult to control the growth of the biofilm mechanically, particularly in individuals with special needs.^1,7,11,23,24,41^ The use of different types of special needs toothbrushes might aid in preventing the occurrence of periodontal disease.^15,26,30^

Contaminated toothbrushes may cause recurrent infections in the oral cavity.^9^ Brushes can harbour microorganisms such as *Streptococcus mutans* that initiate dental caries, *Lactobacillus* bacteria which are responsible for the progression of dental caries, and *Candida albicans* responsible for oral thrush.^27^ Microbes can contact the bristles of a toothbrush via the mouth or through aerosols generated from flushing the toilet, from fingers and hands touching contaminated surfaces, and from microbes residing in damp surroundings.^6^ According to Osho et al^36^ and Harun et al,^18^ the most common microbes found in toothbrushes used two times a day for thirty days were enterococci (10%), *S. aureus,* *S. saprophyticus* (20%) and *Pseudomonas aeruginosa* (40%). Similarly, in another study,^37^ *S. mutans, Klebsiella, Candida, Pseudomonas,* lactobacilli, and *E. coli* were observed. *S. mutans* was the most frequent of all these microorganisms in toothbrushes used 2x/day every day after 1 month.^37^

The present study aimed to compare the efficacies of two unique special needs toothbrushes in plaque removal in Down syndrome patients vs conventional toothbrushes. Additionally, we assessed microbial contamination levels of the two types of toothbrushes after use.

## Materials and Methods

### Study Design and Population

This study was a single-center, randomised, parallel-group study conducted in the Kingdom of Saudi Arabia. The study population comprised sixteen Down syndrome patients (age 6–15 years) randomly chosen from special needs centers located in the Jazan Province of Saudi Arabia and treated at the College of Dentistry, Jazan University. The eligibility criteria were: no history of professional prophylaxis over the past three months; every patient had a caregiver who brushed their teeth two times a day; and no orthodontic banding or removable prosthesis was present. The exclusion criteria were as follows: uncooperative patients; children under medications that affected the gingival/periodontal health; and dental treatment within the past 3 months.

### Informed Consent and Ethical Clearance

The primary objective of this study was briefly described to the subjects (both the children with Down syndrome and their parents/caregivers), and written informed consent was obtained from the patient’s caregivers before the start of the trial. The study was conducted in accordance with the Helsinki Declaration guidelines, and ethical approval was given by the Standing Committee for Scientific Research Ethics at Jazan University (Ref: REC42/1/095).

### Study Protocol

At baseline (T_0_), two principal investigators, calibrated and tested for intra- and inter-examiner reliability, carried out the examination (with a kappa value of 80%). The plaque index and gingival bleeding index (Silness and Löe plaque index; Ainamo and Bay gingival bleeding index)^2,31^ of six teeth (16, 21, 24, 36, 41, and 44) were measured at the initial visit. At the initial visit (T_0_), the parents of the Down syndrome children, who were also the caregivers, were given a conventional toothbrush and instructed to use the correct toothbrushing technique (Fones brushing technique) while brushing their child’s teeth.

Four weeks after the initial visit (T_1_), the plaque index (PI) and gingival bleeding index (BI) of the patients were re-evaluated. Subsequently, the patients were randomly assigned to one of two groups (two different kinds of special needs toothbrushes) via an envelope containing their group number. The envelopes were prepared by a biostatistician using random computer-generated number sequences to conceal the sequence generation and allocation. The envelopes were randomly distributed to the patients by a nurse unconnected to the study who was not aware of its contents. The examiners were blinded to the allocation.

**Fig 1:**
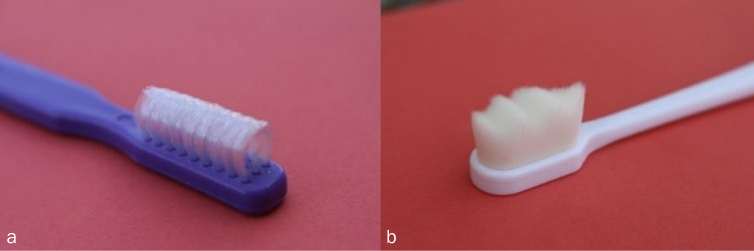
The Collis Curve (a) and superfine nano (b) special needs toothbrushes.

The subjects were assigned into the two groups with an allocation ratio of 1:1 (n = 8, each): one group was given a curved special-needs toothbrush (Collis Curve; Brownsville, TX, USA) and the other group received a superfine nano special-needs toothbrush (Foxvine; Glendale, NY, USA) (Fig 1). Both groups were re-evaluated after four weeks (T_2_). The plaque index and gingival bleeding index were compared between the initial visit (T_0_), second visit (T_1_), and third visit (T_2_).

### Evaluation of Microbial Contamination

Four used special-needs brushes from each group were randomly collected at the T_2_ visit. The brushes were washed in regular tap water and transported to the lab in a sterilised bag. Two-thirds of the bristles from the used toothbrushes were cut using a sterile razor and placed in a test tube loaded with 1-ml Tris-EDTA buffer using sterile forceps. The tube was vortexed for 1 min to break up the bacteria adhered to the surface, and the resultant mixture was cultured in a plate containing Mueller Hinton agar medium. Sterile agar plates were chosen to prepare the agar medium and culture microorganisms with accurate proportions of water and agar powder. Prepared agar medium was poured into the agar plates, which were then stored in the refrigerator to let them cool and prevent contamination. One ml of each of the dilution factors was collected via a sterilized pipette and plated on the plate-count agar. After inoculation, the agar plates were incubated overnight in an incubator at 37°C. After 48 h, the colony-forming units (CFU) on each plate were counted. Universal standardisation of the materials, instruments, methodologies and calibration methods used for the microbial analysis was ensured.

### Statistical Analysis

Data analysis was carried out using SPSS v 20 (SPSS; Chicago, IL, USA). Unpaired Student’s t-tests were used to analyse the results between the Collis Curve and superfine nano special-needs toothbrushes. Paired Student t-tests were used for intra-group comparisons. The statistical significance level was set at 5%.

## Results

**Fig 2:**
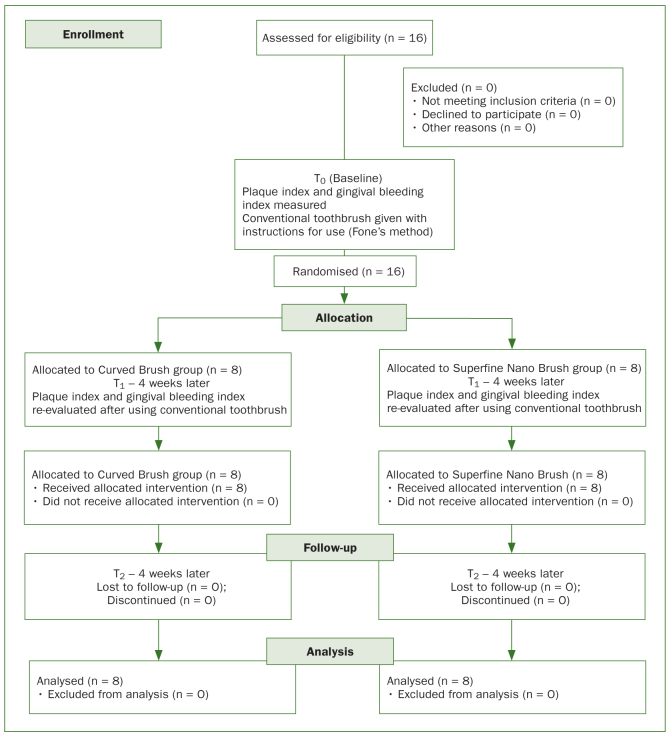
CONSORT flow diagram.

A total of 16 individuals between 6 and 15 years of age were enrolled in the study. Figure 2 depicts the CONSORT flowchart. Table 1 shows the mean plaque index at the initial visit (T_0_, baseline). After using the conventional toothbrush for four weeks, the mean plaque index had decreased in both groups by the second visit (T_1_). These scores showed further improvement with the use of the specially designed toothbrushes after four weeks of use (T_2_). No notable changes in mean plaque index were observed between the two groups at each visit (p > 0.05), but statistically significant reductions were noted between visits in both groups (p < 0.05) (Table 1).

**Table 1:** Plaque index at visits T_0_, T_1_ and T_2_ for Collis Curve and superfine nano toothbrushes

Visit	Collis Curve	p-value	Superfine Nano	p-value	p-value
	Mean ± SD		Mean ± SD		
First visit (T_0_)	82.0 ± 19.4		94.8 ± 12.5		0.141
Second visit (T_1_)	40.0 ± 18.3		46.3 ± 25.1		0.578
Third visit (T_2_)	28.6 ± 12.3		30.4 ± 14.5		0.799

T_0_ – T_01_	42.0 ± 21.7	0.001*	48.5 ± 24.1	0.001*	0.580
T_0_ – T_2_	53.4 ± 22.6	<0.001**	64.4 ± 15.6	<0.001*	0.277
T_1_ – T_2_	11.4 ± 8.9	0.008*	15.9 ± 11.8	0.007*	0.404

*p < 0.05, ** p < 0.001

The mean bleeding index at T_0_ was 69.7 ± 17.5 in the Collis Curve group and 71.1 ± 25.4 in the superfine nano group; this decreased to 38.8 ± 23.6 and 36.8 ± 24.0, respectively, at T_1_ and dropped further to 21.1 ± 17.2 and 21.8 ± 16.1 at T_2_ (Table 2). No statistically significant changes in the bleeding index were seen between the two groups at each visit (p > 0.05); however, statistically significant reductions were noted between visits in both groups (p < 0.05) (Table 2). Overall, both indices showed statistically significant improvement with the use of special needs toothbrushes at T_2_ compared to the regular brushes at T_1_ (p < 0.05).

**Table 2:** Bleeding indices at visits T_0_, T_1_ and T_2_ for Collis Curve and superfine nano toothbrushes

Visit	Collis Curve	p-value	Superfine Nano	p-value	p-value#
	Mean ± SD		Mean ± SD		
First visit (T_0_)	69.7 ± 17.5		71.1 ± 25.4		0.900
Second visit (T_01_)	38.8 ± 23.6		36.8 ± 24.0		0.867
Third visit (T_2_)	21.1 ± 17.1		21.8 ± 16.1		0.935

T_0_ – T_1_	31.0 ± 19.8	0.003*	34.4 ± 31.8	0.018*	0.799
T_0_ – T_2_	48.6 ± 19.1	<0.001**	49.3 ± 30.0	0.002*	0.956
T_1_ – T_2_	17.7 ± 12.2	0.005*	14.9 ± 9.6	0.003*	0.630

*p < 0.05, ** p < 0.001.

**Fig 3:**
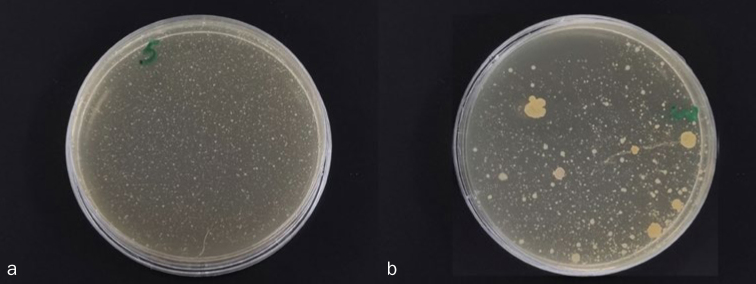
Image showing the bacterial colony forming units (CFU) from the Collis Curve (a) and superfine nano (b) special needs toothbrushes.

The mean number of CFU per plate was 1411.50 in the Collis Curve group and 1118.25 in the superfine nano group (Fig 3). No statistically significant difference was observed between the two special needs toothbrushes with regard to CFU counts (p > 0.05), indicating that both brushes had similar levels of bacterial contamination after use.

## Discussion

Regular dental care and maintenance of oral hygiene are important determinants of oral health in individuals with special needs. Motor impairments can result in limited manual dexterity, making conventional brushing techniques challenging and resulting in ineffective plaque removal.^25,48^ Individuals with developmental and intellectual disorders may require assistance from a caregiver to brush their teeth.^13,33^ Antimicrobials such as chlorhexidine may help in reducing supragingival plaque and control the development of a biofilm.^42^ However, mouthwashes cannot be universally recommended and have limited application in subgingival plaque removal. They are not recommended in young patients or those with poor muscle tone because of their lack of ability to frequently rinse their mouths.^25,48^ Mechanical plaque control through regular brushing and periodic scaling and root planing remain the cornerstone of periodontal therapy. Mechanical disruption of the biofilm is pivotal in preventing the progression of periodontal disease.

The design of a conventional toothbrush especially with regard to its size and contour should be such that it aids in mechanical removal of plaque. ADA specifications for an acceptable toothbrush are with brushing surface length 1–1.25 inches and width 5/16–3/8 inches; surface area: 2.54–3.2 cm^2^; number of bristle rows: 2–4; number of tufts: 5–12 tufts per row; and number of bristles: 80–85 bristles per tuft. For adults, large or medium sized heads would be sufficient. Small brush-heads are recommended for children, as their teeth and mouths are generally smaller.^39^

Research has established the need for customised toothbrushes directed toward special needs children and adults.^49^ This study aimed to clinically evaluate the oral hygiene and gingival health status of patients with Down syndrome using two unique special needs toothbrushes compared to a conventional toothbrush.

We observed that the use of a conventional toothbrush for four weeks resulted in a marked decrease in the mean plaque index (PI) and bleeding index (BI) in both groups. These findings are similar to that reported by Sakellari et al,^38^ wherein marked positive effects of professional scaling and closely monitored dental hygiene practices were observed on the plaque and bleeding on probing indices. The prevalence and the severity of periodontal infection (measured clinically as well as radiographically) in patients with Down syndrome were reduced after proper hygiene measures had been instituted.^38^

In terms of plaque removal, the plaque index scores of both groups improved after using the special needs toothbrushes for four weeks. The results showed that the use of the two different special needs toothbrushes improved the oral hygiene status in terms of plaque and bleeding indices. These toothbrushes have been shown to assist subjects with restricted manual dexterity and are preferred by caregivers who help these patients with toothbrushing. The improvement may be due to the unique design of the brushes. The head of the Collis Curve brush increases the circulation in the gingival area due to its light massaging action, which is suitable for caregivers and individuals with a restricted range of motion (Fig 2). The bristle is curved and flexible and can reach the undercut areas, particularly in malpositioned teeth. The compact head and efficient triple-fit bristles prevent gagging while brushing the posterior teeth and eliminate any harsh poking actions that can occur with a regular straight-bristled brush. It hence appears that brush design per se is more important in dictating the efficiency of plaque removal, and in this regard, both brushes used here were found effective enough in reducing plaque scores. Overall, there is evidence that using customised special-needs toothbrushes can benefit and improve the oral and periodontal health status of individuals with Down syndrome.

The superfine nano toothbrush contains more than 20,000 superfine bristles, which can gently flex and rotate around undercuts and malpositioned teeth. The literature contains only limited data on the effectiveness of customised toothbrushes in Down syndrome patients. A few studies have examined the effectiveness of using a “Digital Brush” in controlling the plaque in healthy subjects.^43,44^ A systematic review by Kalf-Scholte et al^24^ indicated that there was no difference between another type of special needs triple-headed toothbrush compared to one-headed brushes, with a few studies favouring the three-headed toothbrush in the removal of the plaque.

In terms of bacterial contamination, both brushes showed similar amounts of microbial contamination after four weeks of use. Although these differences were not statistically significant, they point to the dangers of storage environments acting as microbial reservoirs. Data from studies using antimicrobially coated toothbrushes also demonstrate significant bacterial contamination rates, as observed in the present study. A study at the University of Alabama stated that toothbrushes stored in the bathroom are extremely likely to have fecal content lingering in their bristles; toilet flushing was shown to produce an aerosol spray of bacteria-tainted water, which could lead to further contamination.^10^ Turner et al^47^ examined the efficacy of chlorhexidine-coated brushes on the reduction of bacterial counts, and found no notable change in the quantities of bacteria between the experimental and the control groups. Al-Ahmad et al^3^ examined the antibacterial impact in-vitro of a toothbrush with silver-coated heads and reported no statistically significant reductions in the number of CFU formed by *S. oralis, S. mutans, S. sanguis, A. viscosus, L. casei*, and *C. albicans.*^3^

One limitation of the present study was the lack of a control group for the conventional manual toothbrush. Moreover, the contamination of the conventional toothbrushes was not assessed. The reason was due to the limited access to Down syndrome patients during the COVID-19 pandemic.

People with disabilities may benefit from regular targeted dental care to improve dental health. The assistance of a caregiver may be invaluable in this regard. However, every effort must be made to ensure that patients are able to self-sufficiently achieve mechanical plaque control through toothbrushing and optimise dental health.

## Conclusions

This study aimed to evaluate the effectiveness of two special needs toothbrushes in plaque control and improving gingival health in patients with Down syndrome. The use of a Collis Curve and superfine nano special-needs toothbrush was found to statistically significantly reduce the plaque index and bleeding index over a period of four weeks. Customised toothbrushes tailored to the requirements of special needs individuals can significantly reduce plaque and bleeding levels compared to conventional toothbrushes.
